# Monitoring Enzyme Activity Using Near-Infrared Fluorescent Single-Walled Carbon Nanotubes

**DOI:** 10.1021/acssensors.4c00377

**Published:** 2024-04-26

**Authors:** Srestha Basu, Adi Hendler-Neumark, Gili Bisker

**Affiliations:** † Department of Biomedical Engineering, Faculty of Engineering, Tel Aviv University, Tel Aviv 6997801, Israel; ‡ Center for Physics and Chemistry of Living Systems, Tel Aviv University, Tel Aviv 6997801, Israel; § Center for Nanoscience and Nanotechnology, Tel Aviv University, Tel Aviv 6997801, Israel; ∥ Center for Light-Matter Interaction, Tel Aviv University, Tel Aviv 6997801, Israel

**Keywords:** enzyme activity, enzymatic reaction, single-walled carbon nanotubes, fluorescence, optical nanosensors

## Abstract

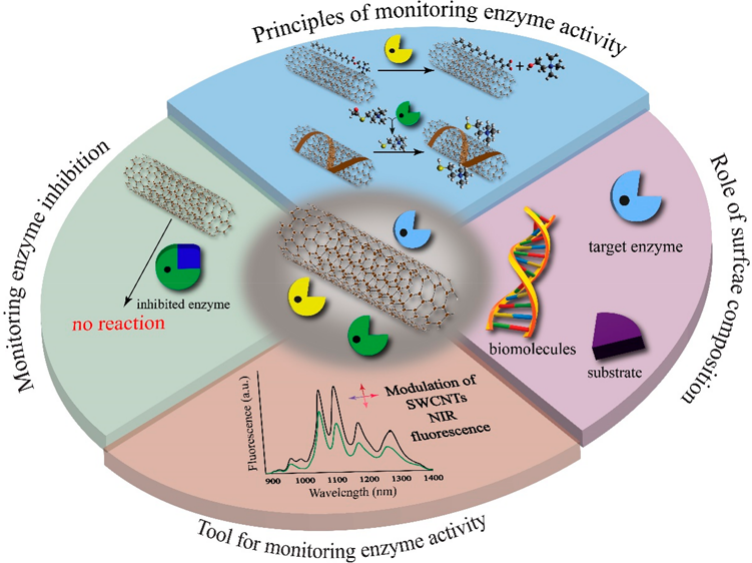

Enzymes serve as pivotal biological catalysts that accelerate essential chemical reactions, thereby influencing a variety of physiological processes. Consequently, the monitoring of enzyme activity and inhibition not only yields crucial insights into health and disease conditions but also forms the basis of research in drug discovery, toxicology, and the understanding of disease mechanisms. In this context, near-infrared (NIR) fluorescent single-walled carbon nanotubes (SWCNTs) have emerged as effective tools for tracking enzyme activity and inhibition through diverse strategies. This perspective explores the physicochemical attributes of SWCNTs that render them well-suited for such monitoring. Additionally, we delve into the various strategies developed so far for successfully monitoring enzyme activity and inhibition, emphasizing the distinctive features of each principle. Furthermore, we contrast the benefits of SWCNT-based NIR probes with conventional gold standards in monitoring enzyme activity. Lastly, we highlight the current challenges faced in this field and suggest potential solutions to propel it forward. This perspective aims to contribute to the ongoing progress in biodiagnostics and seeks to engage the wider community in developing and applying enzymatic assays using SWCNTs.

Enzymes constitute a vital category of biomolecules that accelerate the rate of biochemical processes.^1−3^ The quantification of an enzyme’s effectiveness in biotransformation is achieved by monitoring its activity. The precision with which enzymes function is crucial, as any deviation from their normal activity may be associated with the onset of diseases.^4,5^ Consequently, monitoring enzyme activity provides valuable insights into both health and pathological conditions. Additionally, an investigation into enzyme kinetics and their mechanisms of action is a fundamental component of drug discovery research.^6−9^ Owing to the crucial role played by enzymes in a wide array of biological processes essential for the sustainability of life, tracking enzyme activity offers valuable insights into the mechanistic intricacies of these vital processes. This, in turn, deepens our comprehension of fundamental biological processes. Hence, the monitoring of enzyme activity emerges as a crucial task for gaining a deeper understanding of cellular processes,^10,11^ thereby exerting a substantial influence in various applications, including drug screening,^12^ point-of-care diagnostics,^13,14^ and bioremediation,^15,16^ among others.

Notably, numerous endeavors have been undertaken to monitor enzyme activity, employing a variety of luminescent probes predominantly operational in the UV–vis region.^17−20^ For example, the activity of numerous enzymes has been tracked through fluoroimmunoassays, utilizing fluorescently labeled antigens and antibodies.^21^ In typical assays of this nature, fluorescently labeled antigens are displaced from antibody binding sites by label-free products generated from enzymatic reactions. These results show significant changes in the fluorescence of the antigen–antibody complex. However, these diagnostics come with inherent limitations, such as assay heterogeneity, slow reaction kinetics, and the necessity for multiple incubation and washing steps. Additionally, the fluorogenic substrates often consist of aromatic dyes that may competitively bind to the enzymes and are susceptible to photobleaching under extended light irradiation.^21^ Therefore, substantial efforts have been dedicated to substituting fluoroimmunoassays with alternative assays that utilize chemosensor-based monitoring of enzyme activity. In that regard, fundamental principles of fluorescence have been harnessed as tools to craft suitably designed chemosensors for the monitoring of enzyme activity. For example, chemically interactive sensors operational on the basis of Förster resonance energy transfer (FRET) have been reported to successfully detect the activity of a variety of enzymes.^22^ Moreover, the fluorescence modulation of chemosensors upon selective interaction with either the substrates or the products of enzymatic reactions has also been demonstrated as a straightforward strategy for screening enzyme activity.^23^

While these methods are effective for investigating enzyme activity, it is important to acknowledge that they might not be the optimal choice for real-time monitoring of enzyme activity, especially within biological fluids or whenever real-time spatiotemporal information is needed.^24−26^ Limitations associated with readouts in the UV–vis spectral region include factors like autofluorescence emitted by biological entities such as cells, tissues, blood, and plasma.^27,28^ For example, various biological entities, such as hemoglobin, exhibit distinctive spectral features, such as the Soret band, which closely aligns with the absorption peak resulting from the enzymatic hydrolysis of acetylthiocholine by acetylcholinesterase. This alignment can lead to background interference in the enzyme assay.^29^ In light of these considerations, the significance of luminescent probes operating in the near-infrared (NIR) region becomes evident. These probes offer real-time and spatiotemporal information, and their utility is particularly notable because biological samples are mostly transparent in the NIR region.^30−37^ In pursuit of this objective, NIR luminescent single-walled carbon nanotubes (SWCNTs) are advantageous due to their intrinsic physicochemical and optical properties.^28,38−46^ These include the high surface area and the ease of surface functionalization with molecules of choice that facilitate interaction with a large number of analytes, enabling high-throughput readouts.^47−56^ Moreover, SWCNTs exhibit remarkable photo and chemical stability, rendering them a compelling choice as sensors for monitoring essential biomarkers, including proteins,^57−66^ oncometabolites,^67^ pathogens,^68,69^ various small molecules,^70−77^ hormones,^58,78,79^ volatiles,^80,81^ lipids,^44,82,83^ sugars,^84−89^ neurotransmitters,^30,34,90−94^ microRNA,^95,96^ metal ions,^97^ lysosomal pH,^98^ and enzymatic activity and inhibition.^99−107^

Taking advantage of these attributes, SWCNTs have been employed in the monitoring of enzymatic activity through multimodal approaches. In some studies, appropriately functionalized SWCNTs probed the product of enzyme activity,^99^ while in others, SWCNTs were dispersed with the substrate of a chosen enzyme.^104^ The resultant chemical transformations were transduced into a modulation of the fluorescence signal, effectively facilitating enzyme monitoring in real-time. In specific studies, enzyme inhibition was examined by synthetically impeding the enzymes such that no fluorescence modulation of the SWCNTs was observed.^99^ Conversely, in other studies, enzyme inactivation was probed by fluorometric detection of the product generated in the inactivation pathway.^102^ Moreover, in some pursuits, the surface of SWCNTs has been modified to interact with reactants and products of enzymes, thereby not only allowing facile monitoring of enzymatic activity, but also enabling signal amplification vis-à-vis conventional techniques.^103^ Importantly, in certain studies, it has been explicitly demonstrated that SWCNTs-based detection of enzyme activity is comparable to, or even superior to, conventional assays in terms of sensitivity. Furthermore, it has been emphasized that SWCNTs-based optical transducers outperform conventional probes for monitoring enzymatic assays due to their optical and temporal stability in biological media, consistent dynamic range of response, reduced susceptibility to interfering molecules, and minimized background interference.^99,103,104,106,107^ Thus, recognizing the broad significance of enzymes and the progress made in utilizing SWCNTs in this context, it is imperative to delineate the essential features of studies conducted to monitor enzyme activity using NIR luminescent SWCNTs.

Here, we first aim to identify the fundamental properties of SWCNTs that render them well-suited for enzyme monitoring and explore diverse strategies for assessing enzyme activity and inhibition, with a particular emphasis on the chemical interactions that enable such effective monitoring. We further discuss the apparent advantages of monitoring enzyme activity using SWCNTs, underscoring the generality of the underlying principles, and provide key insights and design principles for future studies in this domain. Finally, we address the existing challenges in this research field and put forth potential solutions to propel further advancements.

## SWCNTs as Optical Probes for Monitoring Enzyme Activity

Ensuring adherence to certain essential criteria is crucial when selecting a specific probe for biomarker monitoring, and tracking enzyme activity is no exception. Therefore, before delving into the various approaches used to monitor enzyme activity with SWCNTs, it is important to identify the key physicochemical and optical properties that make them well-suited for such applications.

A fundamental characteristic of SWCNTs that enhances their suitability for biosensing applications is their well-defined surface, which can be functionalized with chosen molecules using a repertoire of principle chemistry.^108−112^ This tailorable chemical versatility provides options for monitoring enzyme activity, either through the direct interaction of molecules tethered onto the surface of the SWCNTs with enzymes or by detecting the products of enzymatic reactions. Further, the role of the SWCNT surface is particularly crucial in the case of monitoring enzyme activity, given that the high surface area of SWCNTs provides multiple sites for enzyme–substrate interactions, enabling a high-throughput readout.^84^ Additionally, the surface functionalization of SWCNTs significantly influences properties such as their dispersibility in biologically relevant media and, consequently, their biocompatibility.^113^ The biocompatibility of a probe is particularly crucial for the continuous monitoring of any biological process. In this context, it is noteworthy that SWCNTs, following appropriate surface functionalization, can be rendered biocompatible,^114,115^ making them further well-suited for monitoring enzyme activity. Moreover, through proper functionalization, SWCNTs can be stabilized in biological media without significant biodegradation,^37^ offering an option for the *in vivo* monitoring of enzyme activity.

Additionally, SWCNTs exhibit discernible fluorescence in the near-infrared spectral region, where most biological entities, such as tissues, blood, and plasma, are predominantly transparent.^27,116−119^ This renders SWCNTs suitable for monitoring enzyme activity in clinical samples with minimal background interference. Furthermore, the exceptional stability of SWCNTs under continuous light irradiation, without experiencing photobleaching or blinking, makes them suitable for the prolonged monitoring of enzyme activity within biological media.^120^ Importantly, SWCNTs offer high spatiotemporal resolution, providing distinct advantages over conventional bulk measurements.^52,121,122^ This attribute makes them particularly well-suited for screening enzyme activity with a high degree of specificity. Lastly, the fluorescence of SWCNTs is highly dependent on their surface composition.^55,123^ Consequently, enzymatic reactions causing any alterations in the surface composition of SWCNTs are directly transduced into optical signals manifested in the modulation of the emitted fluorescence, enabling highly sensitive monitoring of enzyme activity. Due to these virtues, SWCNTs may be considered an ideal candidate for probing enzyme activity.

## Approaches for Monitoring Enzyme Activity

Having established the suitability of SWCNTs as probes for monitoring enzyme activity, we aim to underscore the various strategies that have been employed for this purpose in this section.

### SWCNTs Suspended by the Substrate of the Target Enzyme

#### Substrates as the *Primary* Dispersant of SWCNTs

**Scheme 1:**
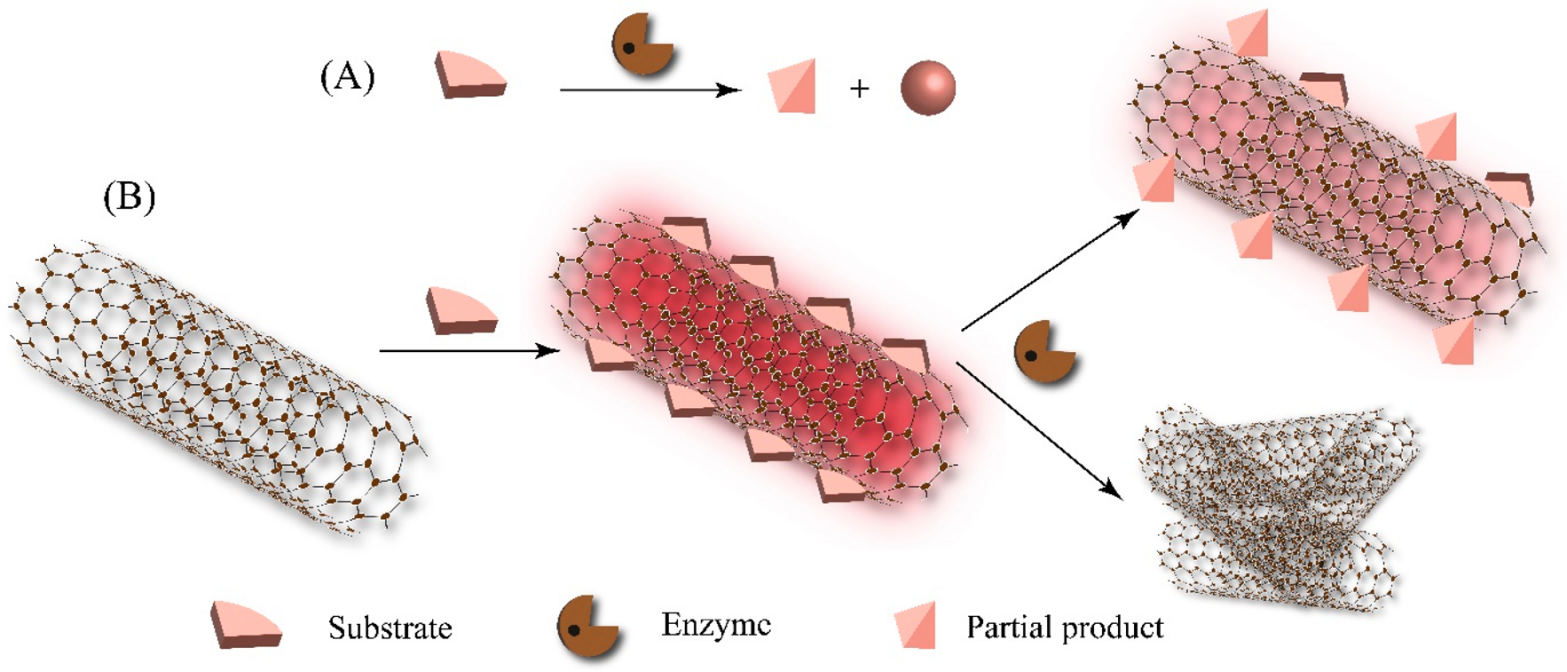
Schematics of the Principles for Monitoring Enzyme Activity Using Substrate-Suspended SWCNTs: (A) Illustration of an Enzymatic Reaction Transforming a Substrate into a Product and a Byproduct and (B) the Substrate Used for Suspending the SWCNTs Subsequently, the enzymatic activity can alter the surface composition of the substrate-suspended SWCNTs, resulting in a modulation of the SWCNT NIR fluorescence. Alternatively, the enzymatic activity may partially or completely displace the substrates from the SWCNT surface, triggering their aggregation, which consequently leads to fluorescence quenching.

As previously mentioned, the predominant feature of SWCNTs that enhances their suitability for enzyme activity monitoring is their surface availability for functionalization. Consequently, the effective functionalization of the SWCNT surface plays a crucial role in successfully utilizing SWCNTs as probes for monitoring enzyme activity. A straightforward approach in this line involves suspending the SWCNTs with the substrate of the enzyme, whose activity is under assessment. In this process, the chemical composition of the substrate should possess two essential functional components, namely a hydrophobic group for binding and covering the SWCNT surface, and a hydrophilic group to facilitate the dispersion of SWCNTs in an aqueous medium. After suspending the SWCNTs with the substrate, they can be exposed to the target enzyme for a necessary duration, enabling enzymatic activity to take place. The anticipated outcome is that the enzyme activity would cleave or transform the substrate attached to the SWCNT surface, resulting in a modification of the surface composition. This alteration can then be transduced into an optical signal, such as a change in the fluorescence intensity or a shift in the peak emission wavelength, thereby allowing effective monitoring of enzyme activity using SWCNTs as probes. Another possibility in this context could be the partial or complete removal of the substrates from the surface of the SWCNTs following enzyme activity, thereby causing aggregation of the SWCNTs, leading to a decrease in their fluorescence (Scheme 1).

**Figure 1:**
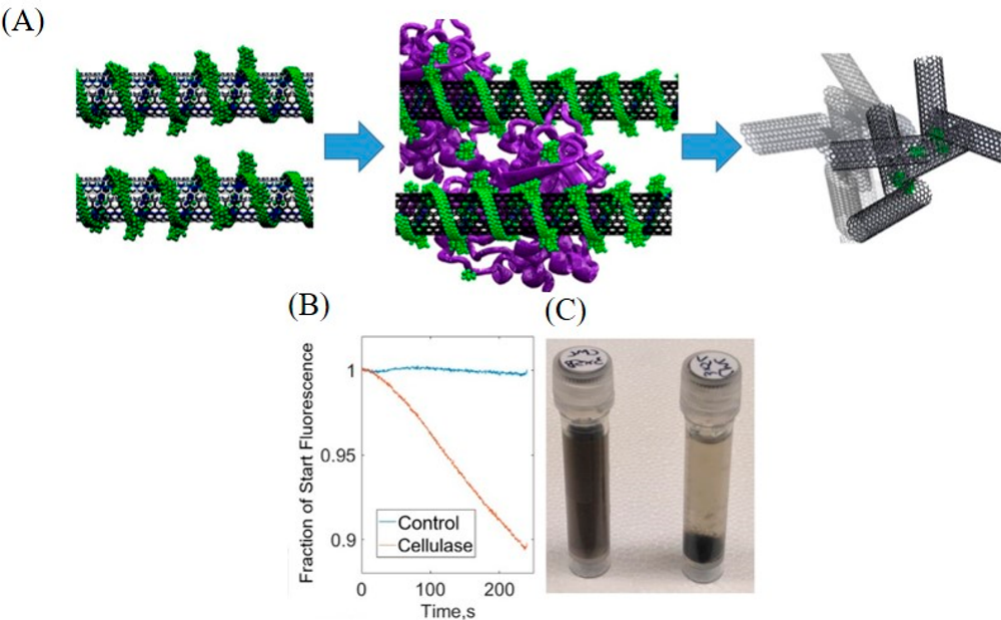
(A) Schematic illustrating the principle of monitoring enzyme activity through aggregation-induced quenching of the fluorescence in substrate-wrapped SWCNTs. (B) Time-dependent decrease in the fluorescence of CMC-suspended SWCNTs following cellulase activity. (C) Digital photographs showing aggregation of CMC-wrapped SWCNTs following cellulase activity. Reproduced with permission from ref (104). Copyright 2018 American Chemical Society.

In this regard, the Reuel research group has shown that amphiphilic substrates of hydrolytic enzymes, when encased around (6,5) SWCNTs, could react to the hydrolytic enzyme’s activity, offering real-time insights into the enzyme concentration and potential damage inflicted on the enzyme.^104^ The stabilization of (6,5) SWCNTs was achieved using various substrates such as carboxymethylcellulose (CMC), pectin, and BSA, corresponding to their respective hydrolytic enzymes—cellulase, pectinase, and bacterial protease. The enzymatic activity resulted in the gradual degradation of the substrates, ultimately leading to the diminishing intensity of the SWCNT fluorescence, primarily attributed to their aggregation. This study, being highly specific, further opens new avenues for real-time monitoring of enzyme activity in real-world biological specimens (Figure 1). Further, it has been shown that incubation of the substrate appended SWCNTs with thermally denatured enzymes did not lead to a discernible change in the optical signal of the SWCNTs, thereby validating that the sensor response was exclusive to the enzyme activity and not matrix elements present in the sample. Additionally, SWCNTs suspended with albumin, CMC, and lignosulfonic acid (LSA) were utilized to detect the activity of soil enzymes.^124^ This represents an advanced application in monitoring the activity of enzymes using SWCNTs in contexts relevant to industrial prosperity. Furthermore, the Reuel group also utilized biofilm extracellular polymeric substances (EPS) suspended SWCNTs to assess the activity of hydrolase enzymes on EPS.^125^ The degradation of biofilm EPS, which formed the wrapping around the SWCNTs, occurred upon the activity of hydrolase. This degradation was manifested and quantified through a decrease in the fluorescence intensity of the SWCNTs.

Further, our laboratory has exemplified such an approach, wherein the surface of SWCNTs was strategically functionalized with myristoylcholine (MC), serving as the substrate for cholinesterase (CHE) enzymes.^107^ MC is a condensation product of myristic acid and choline, comprising a lengthy hydrocarbon chain with 14 carbon atoms coupled with a positively charged choline group. Importantly, upon hydrolysis by CHE, MC molecules undergo cleavage into myristic acid and choline. Two critical attributes of MC guided the choice to suspend SWCNTs with MC, including its chemical composition, characterized by the simultaneous presence of a long hydrocarbon chain and a hydrophilic group, and its susceptibility to hydrolysis by CHE. This approach was anticipated to induce a suitable modulation in the fluorescence of SWCNTs due to the alteration in their surface composition upon CHE hydrolysis.

**Figure 2:**
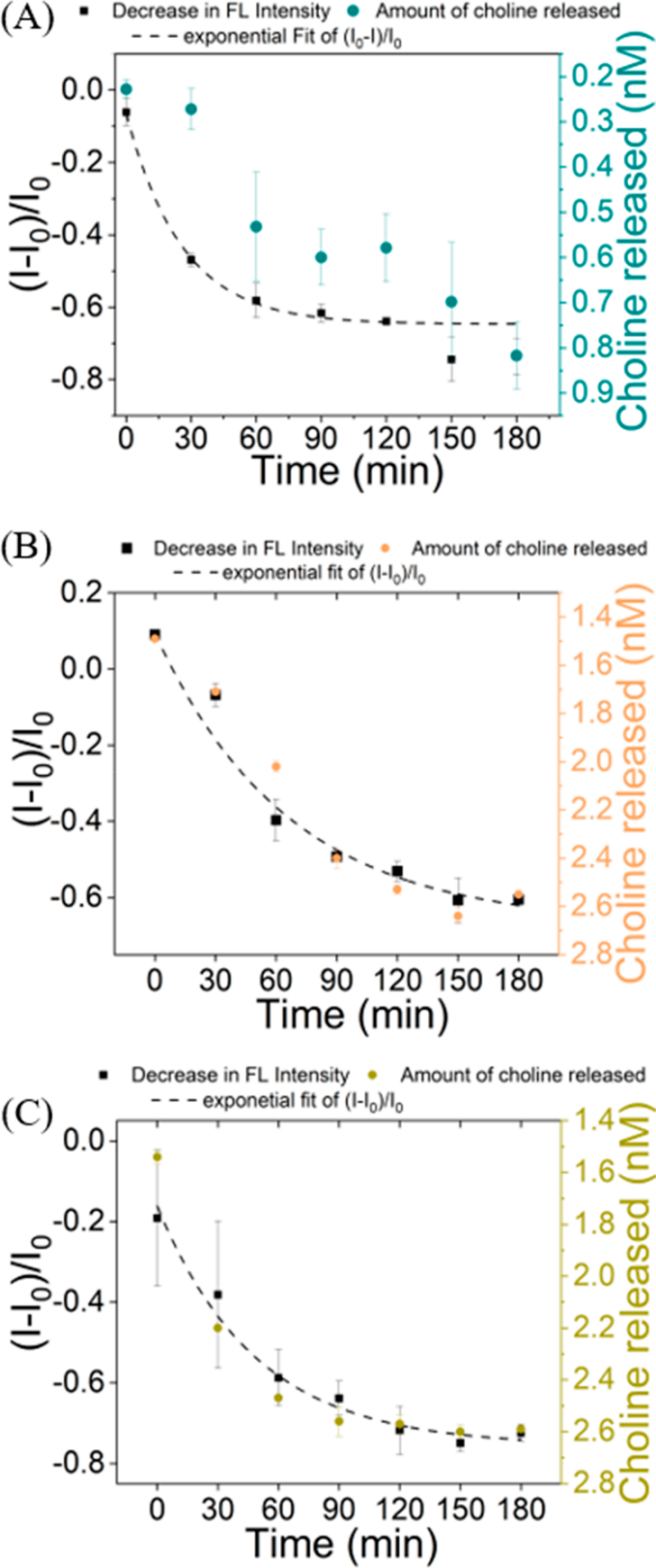
(A) Correlation between the time-dependent normalized fluorescence intensity of MC-SWCNTs upon exposure to ACHE and the amount of choline released as a function of ACHE activity. (B) Correlation between the time-dependent normalized fluorescence intensity of MC-SWCNTs upon exposure to BCHE and the amount of choline released as a function of BCHE activity. (C) Correlation between the time-dependent normalized fluorescence intensity of MC-SWCNTs upon exposure to P-CHE, and the amount of choline released as a function of P-CHE activity. Reproduced with permission from ref (107). Available under a Creative Commons CC BY license. Copyright 2024 John Wiley and Sons.

Incubating MC-SWCNTs with acetylcholinesterase (ACHE), butyrylcholinesterase (BCHE), and the cholinesterase (CHE) found in human blood plasma (P-CHE)—predominantly in the form of BCHE—resulted in a discernible decrease in the fluorescence intensity of MC-SWCNTs over time. To attribute this decrease in fluorescence of MC-SWCNTs when exposed to CHE enzymes, an orthogonal choline assay was conducted to estimate the amount of choline released over time. Interestingly, the quantity of choline released exhibited a strong correlation with the extent of fluorescence decrease of MC-SWCNTs observed in the cases of ACHE, BCHE, and P-CHE. This allowed us to unambiguously attribute the decrease in the fluorescence signal of MC-SWCNTs to the hydrolysis of MC molecules constituting the dispersant of the SWCNTs (Figure 2). Also, the sensitivity of this strategy in monitoring CHE activity was observed to be parallel to that achieved by conventional gold standards like the Ellman assay.^126^

The merit of suspending SWCNTs with the substrates of target enzymes for monitoring enzyme activity lies in several factors, including the simplicity of the approach, direct monitoring of enzyme activity (without the involvement of additional agents), and its high specificity toward the activity of the enzyme of interest. The exclusion of interference from background analytes in fluorescence-based enzyme activity monitoring is a notable advantage attributed to the augmented specificity of substrate-suspended SWCNTs toward the enzyme reactions. This characteristic is evidently the reason for the applicability of this approach in real-time monitoring of enzyme activity in complex biological fluids, such as plasma. This capability undoubtedly paves the way for using substrate-suspended SWCNTs as optical probes for point-of-care diagnostics. While the aforementioned strategy has proven effective, it is important to recognize that this approach may not be universally applicable, as not all substrates for target enzymes possess naturally occurring functional groups able to suspend SWCNTs as a primary or secondary dispersing agent around them. This scenario might necessitate appropriate chemical modification of the substrate in order to implement this enzyme activity detection strategy.

#### Substrates as the *Secondary* Dispersant of SWCNTs

An alternative strategy for monitoring enzyme activity involves grafting the substrates to the surface of prefunctionalized SWCNTs, thereby forming a secondary dispersant around the nanotubes. Subsequent reactions with the enzyme induce substrate transformation, forming products that further interact with the SWCNTs. This interaction then leads to surface modification of the SWCNTs, translating to modulation of their fluorescence and enabling effective monitoring of enzyme activity. For effective monitoring, it is crucial that the product of the enzyme activity can bind with the SWCNTs, leading to discernible fluorescence changes. This strategy is envisioned to not only allow real-time monitoring of enzyme-mediated substrate transformation but also facilitate tracking stepwise interactions of products with SWCNTs.

**Figure 3:**
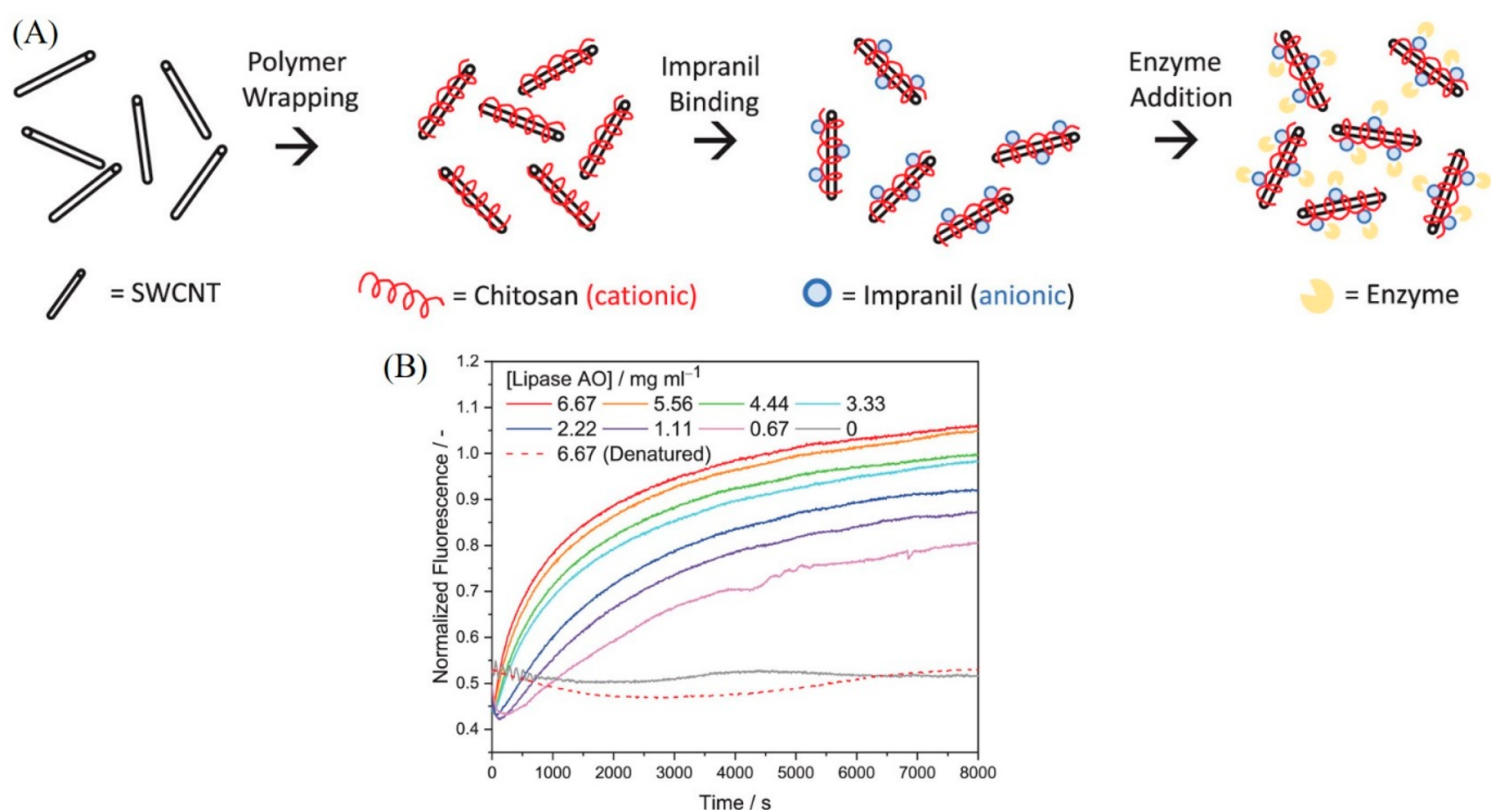
(A) Schematic of the strategy used for screening the activity of lipase (enzyme) using Impranil (substrate) tethered-chitosan stabilized SWCNTs. (B) Time-dependent change in the fluorescence of Impranil (substrate) tethered-chitosan stabilized SWCNTs upon incubation with active and denatured lipase. Reproduced with permission from ref (106). Copyright 2023 American Chemical Society.

To this end, the Reuel research group monitored the degradation of Impranil, a substrate representing a class of polyester polyurethane nanoparticles, by lipase using chitosan-stabilized SWCNTs as NIR-emitting probes.^106^ The negatively charged Impranil particles were proposed to bind to the SWCNTs through electrostatic interaction with the positively charged chitosan-defined dispersant of the SWCNTs. Upon incubation with lipase, Impranil-attached SWCNTs exhibited fluorescence quenching at shorter reaction times, followed by brightening of the fluorescence of the SWCNTs at relatively longer reaction times (Figure 3). This phenomenon was explained based on the proposition that the degradation of Impranil particles from the surface of the SWCNTs led to a decrease in their fluorescence, whereas binding of the degraded Impranil particles to the SWCNTs resulted in a subsequent increase in the fluorescence of the SWCNTs.

This strategy becomes particularly important for substrates undergoing stepwise transformations. The key advantage of this technique lies in its ability to provide a deeper insight into the mechanistic aspects of the enzymatic process through the sequential optical signal modulation of the SWCNTs. Consequently, this method opens additional opportunities for quantitative measurement of key parameters of enzyme kinetics using the fluorescence alteration of SWCNTs as inputs to classical models, such as the Michaelis–Menten model. Such pursuits are likely to represent an important advancement in the field of monitoring enzyme kinetics using NIR-emitting SWCNTs.

**Figure 4:**
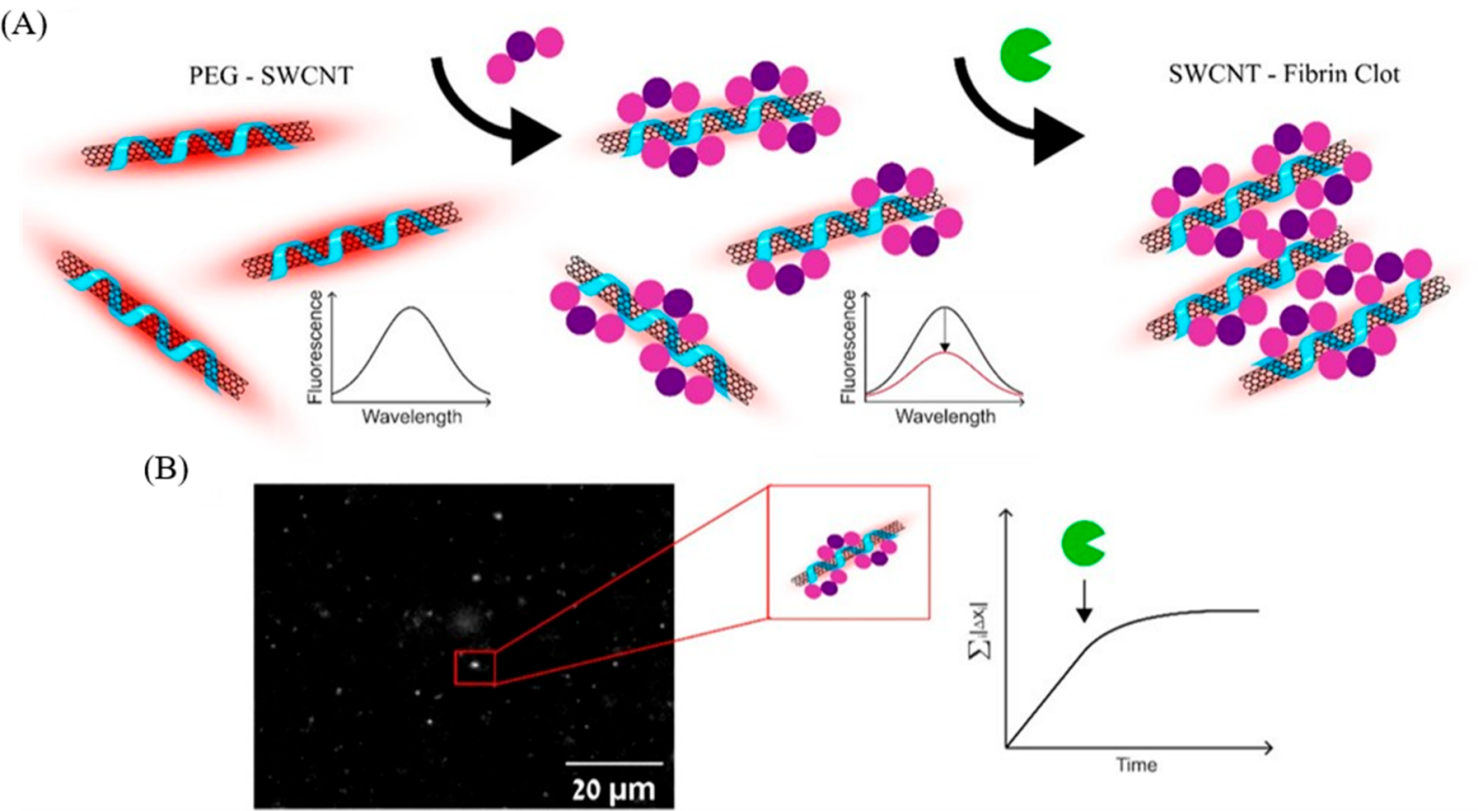
(A) Schematic depicting the binding of fibrinogen to DPPE-PEG-SWCNTs, leading to a decrease in the SWCNT fluorescence and a subsequent thrombin-mediated formation of the fibrin clot encapsulating the SWCNTs. (B) Single-particle tracking of individual NIR-fluorescent DPPE-PEG-SWCNTs during clot formation reveals a gradual deceleration in SWCNT diffusion following the introduction of thrombin. Reproduced with permission from (100). Copyright 2023 American Chemical Society.

In the context of employing substrates as secondary dispersants for SWCNTs for monitoring enzyme activity, our laboratory has developed a technique to monitor the formation of fibrin clots from fibrinogen mediated via thrombin in the NIR region using SWCNTs as optical probes.^100^ As a starting point, SWCNTs were functionalized with dipalmitoylphosphatidylethanolamine-polyethylene glycol (DPPE)–PEG. Subsequently, fibrinogen was bound to the functionalized SWCNTs based on a previously reported observation where DPPE–PEG SWCNTs were found to detect fibrinogen through concomitant binding of the latter to the SWCNTs.^47^ In the next step, thrombin, a serine protease-based enzyme, was added to fibrinogen-appended DPPE-PEG SWCNTs. This gradually led to the conversion of fibrinogen to insoluble fibrin through the polymerization of monomeric fibrins, forming a fibrin clot incorporating the SWCNTs (Figure 4a). Importantly, the diffusion rates of SWCNTs within the fibrin clots were found to slow down upon gradual clot formation, which was contingent on the concentration of active thrombin and the concentration of clottable fibrinogen, thereby serving as a tool not only for monitoring the activity of thrombin or the conversion of fibrinogen to fibrin, but also for obtaining real-time spatiotemporal information on fibrin clotting process, which is a crucial aspect of the coagulation cascade (Figure 4b).

The uniqueness of this strategy for monitoring enzymatic activity is rather straightforward, as it has already been demonstrated to have a distinct application in screening biologically relevant processes mediated by enzymes. Moreover, the success of this strategy and its applicability toward monitoring critical enzyme-mediated biological processes stemmed from the fact that the SWCNTs embedded within a fibrin clot featured different rates of diffusion compared to the diffusion rates of DPPE-PEG SWCNTs bound to fibrinogen before the addition of thrombin. Thus, this methodology can be extended to monitor enzyme-catalyzed biological processes leading to confinement of SWCNTs within insoluble polymeric species, eventually leading to variation in their diffusion kinetics.

Despite the common characteristic between the aforementioned studies, wherein the substrates formed the secondary dispersant phase, it is important to note that the mechanisms for monitoring enzyme activity in the two cases differ. In the detection of thrombin activity, the principle relied on variations in the diffusion rate of SWCNTs within fibrin clots formed upon thrombin-mediated conversion of fibrinogen to fibrin. Conversely, in lipase activity detection, the principle relied on the binding of the substrate in both nondegraded and degraded forms to the SWCNT, leading to discernible changes in the fluorescence of the SWCNTs.

### Tailored Dispersant Functionalization of SWCNTs

The dispersant of SWCNTs plays a critical role in defining a majority of their physical and chemical properties, thereby influencing the SWCNT sensing potential. A challenge arises, however, considering that not all enzymes have clearly defined substrates capable of interacting with the SWCNT surface to facilitate their suspension. Often, the necessary functional groups required for suspending the SWCNTs are missing in these substrates. Addressing this issue involves identifying potential dispersants of SWCNTs that can be chemically modified to incorporate the necessary functional groups. By adding the requisite functional groups, SWCNTs can be suspended effectively and be made responsive to specific target enzymes. This approach offers a versatile strategy for enzyme activity monitoring.

Amphiphilic chemical moieties are vital in this framework. Given the inherent hydrophobicity of all-carbon SWCNTs, ensuring their suspension in aqueous environments is essential, especially given the biological contexts of their use. Equally important as amphiphilicity is the requirement that the amphiphilic molecules can also be chemically modified to incorporate the recognition site of the target enzyme. In essence, developing a universal chemical platform that can be tailored through chemical modifications to introduce functional groups allowing for the suspension of SWCNTs and serving as the enzyme recognition site is a crucial step toward developing generalized strategies for monitoring enzyme activity.

**Figure 5:**
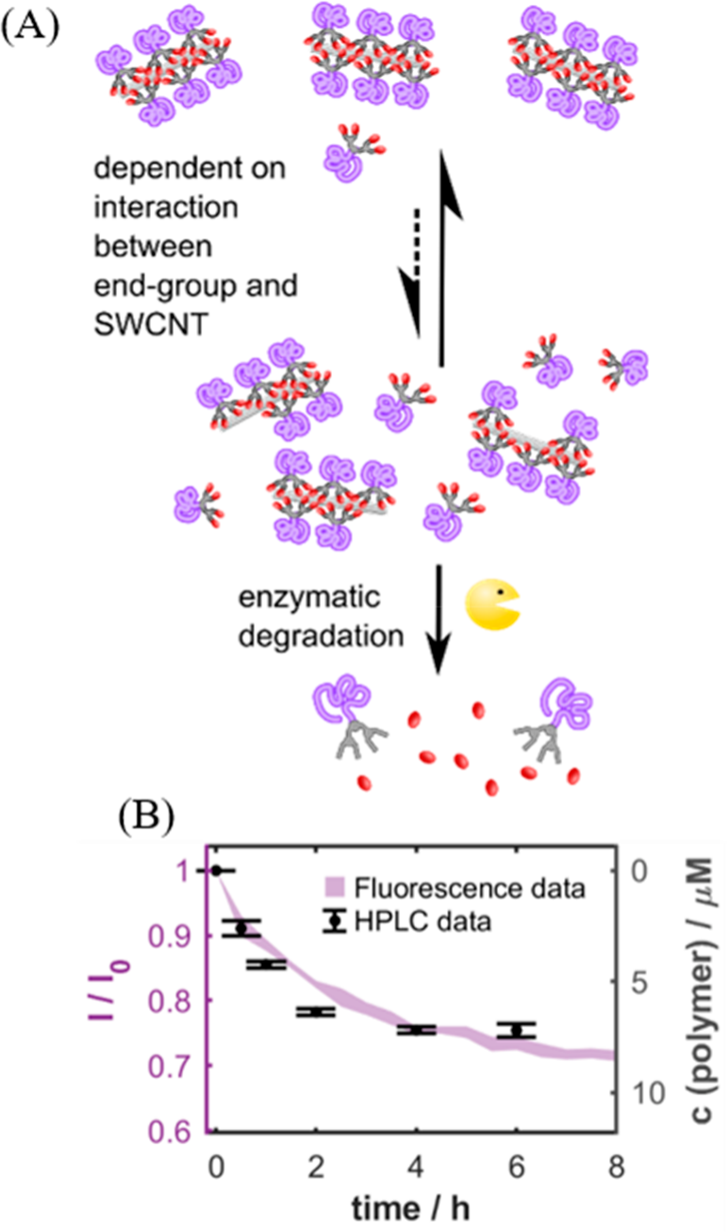
(A) Schematic highlighting the degradation of PEG–dendron hybrid suspended SWCNTs via esterase activity. (B) Correlation between the time-dependent change in the fluorescence of SWCNTs comprising of D-hexanoate and the concentration of end groups released measured by HPLC, as a function of esterase activity. Reproduced with permission from ref (101). Copyright 2021 American Chemical Society.

To achieve this, the synthesis of polymer–dendron hybrids, acting as versatile amphiphilic agents for suspending SWCNTs in aqueous media, has been reported.^101^ The significant advantage of incorporating dendrons as functional components in the composite designed for monitoring enzyme activity arises from the abundant spatial availability of the dendritic end groups to interact with the surface of SWCNTs. The hybrid structure, thus designed from polymers and dendrons, comprised three fundamental structural components: (1) a linear polymeric chain, which was polyethylene glycol (PEG) in our case and functioned as the hydrophilic block, (2) the dendron body contributing to the hydrophobic block, and (3) hydrophobic end groups covalently linked to the dendrons through functional moieties such as ester and amide bonds that were prone to hydrolysis by enzymes like esterase and amidase, respectively. When SWCNTs suspended with the PEG–dendrons hybrid, which included ester or amide groups, were exposed to esterase or amidase, respectively, hydrolytic cleavage of the corresponding bond occurred, leading to an alteration in the surface composition of the SWCNTs, which was transduced into a modulation of the SWCNT fluorescence intensity. An orthogonal assay utilizing high-performance liquid chromatography (HPLC) was conducted to track the release of the end groups following enzymatic activity, revealing a correlation between the decrease in the SWCNT fluorescence and the enzymatic degradation of the PEG–dendrons present as the SWCNT dispersant (Figure 5).

The distinct advantage of this approach, rendering it generically applicable for monitoring enzymatic activity under robust conditions, stems from the facile chemical and structural tunability of the dendrons that integrate the recognition sites for target enzymes. With synthetic control over the hydrophobic end groups in polymer–dendron hybrids, it becomes feasible to deterministically incorporate specific functional groups for monitoring the activity of a desired enzyme. This envisages the potential to monitor a broad class of enzymes, establishing this approach as a versatile platform.

While this strategy holds promise as a generic approach for monitoring enzyme activity, it may encounter some challenges. The use of synthetically engineered substrates to suspend the SWCNTs, which may differ from the naturally occurring substrate, ultimately results in monitoring enzyme activity on proxy substrates rather than on natural ones. In some cases, such proxies may not fully represent the real-world complexity of the actual substrates,^127^ thus potentially compromising the full realization of enzyme activity during screening processes.

### Detecting the Product of Enzyme Activity

Monitoring of enzyme activity products, through changes in the optical properties of appropriate transducers, especially in the NIR region, provides a unique approach for screening enzyme activity. In this approach, appropriately functionalized SWCNTs are incubated with the substrates of a specific enzyme alongside the enzyme itself. It is crucial that the SWCNTs do not directly bind with the substrate or the enzyme, or if they do, such interactions should not be transduced into observable optical signals. Under these ideal conditions, the enzymatic reaction chemically transforms the substrate into designated products, subsequently interacting with the SWCNTs and leading to a substantial alteration in their fluorescence. This approach allows for effective enzyme activity monitoring without requiring synthetically involved dispersant.

**Figure 6:**
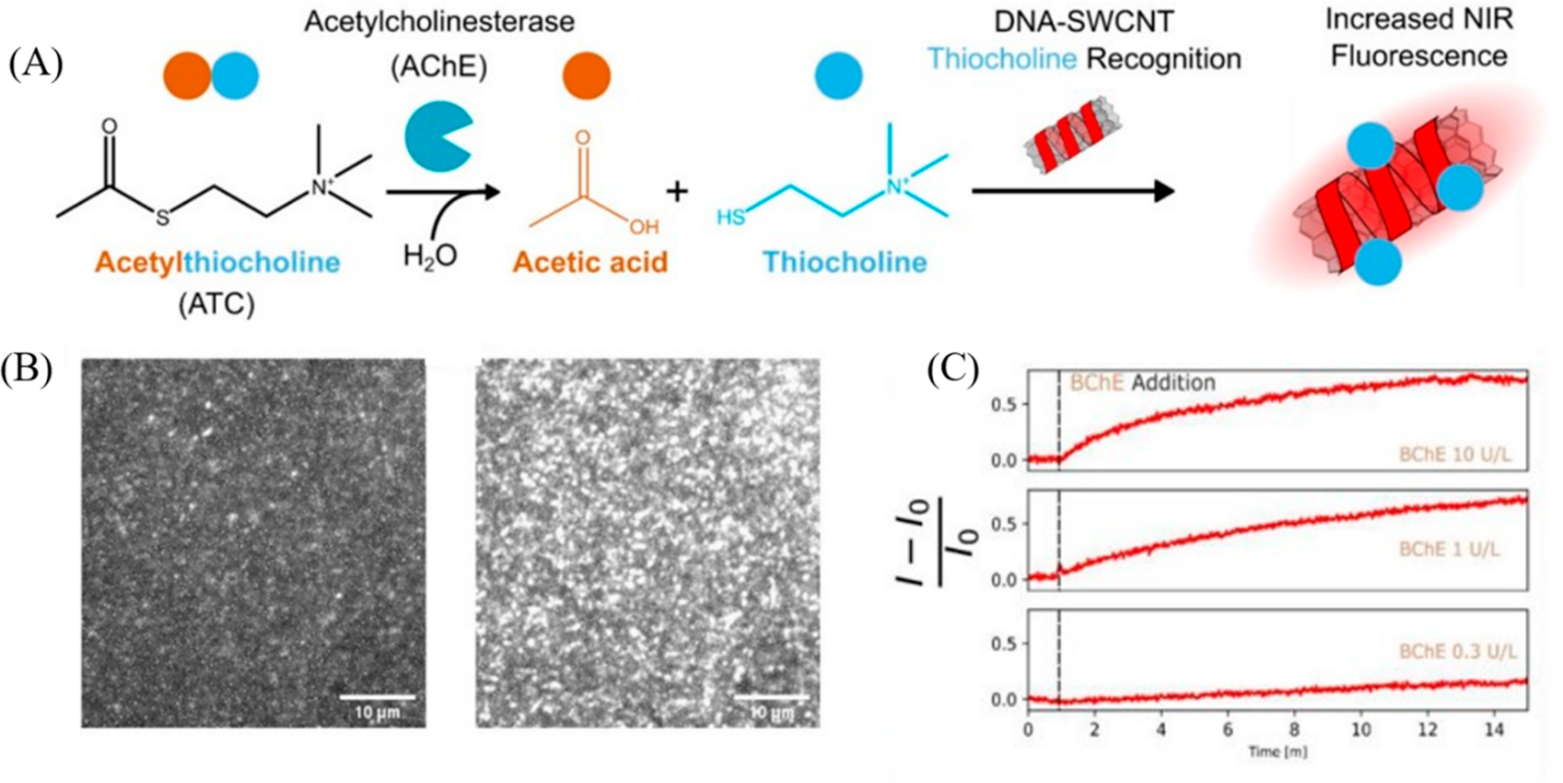
(A) Schematic of the principle developed for monitoring the activity of ACHE by following the formation of TC using DNA-suspended SWCNTs. (B) NIR fluorescence images of SWCNTs before (left) and after (right) the addition of BCHE. (C) Change in the normalized fluorescence intensity of individual DNA-SWCNTs as a function of time for varying BCHE activity. Reproduced with permission from ref (99). Copyright 2022 American Chemical Society.

In this scenario, monitoring of ACHE and BCHE activity using DNA-functionalized SWCNTs by probing thiocholine (TC), the product of cholinesterase-mediated hydrolysis of acetylthiocholine (ATC), was demonstrated.^99^ Following a library screening of DNA-suspended SWCNTs, including (GT)_15_-, (T)_30_-, (TAT)_4_-, (GTTT)_7_-, and (GC)_30_-SWCNTs, (GT)_15_-SWCNTs and T_30_-SWCNTs were identified as ideal sensors for monitoring the enzymatic activity of ACHE, as they reacted to the product of ATC hydrolysis upon enzymatic reaction with ACHE, featuring selective fluorescence enhancement, while remaining unresponsive to the substrate or the enzyme itself (Figure 6a). To further demonstrate the selectivity of the chosen sensors to TC, (GT)_15_-SWCNTs and T_30_-SWCNTs were exposed to various small molecules such as cysteine, choline, acetic acid, and Neostigmine (NE, an inhibitor of ACHE). Interestingly, while under identical conditions, TC led to a significant increase of the fluorescence intensity of the SWCNT sensors, the control molecules resulted in minimal to negligible fluorescence change. Additionally, the applicability of the sensors was demonstrated in a serum environment, further substantiating the robustness of the sensors in a complex biological scenario. The generality of the principle was further demonstrated by monitoring the activity of BCHE, also in complex biological environments like fetal bovine serum, thereby highlighting the real-world applicability of this technique. Notably, enzyme activity could be detected at a single SWCNT level using this approach, providing spatiotemporal resolution of CHE activity in the NIR region (Figure 6b, c).

The primary advantage of this approach in monitoring enzyme activity lies in its capability to provide a direct tool for tracking the formation of the product of enzymatic reactions. While enzymes operate with high specificity in the biological regime, significant deviations from their specificity can occur depending on substrate concentration, reaction conditions, and the presence of other molecules.^128^ Additionally, genetic mutations can alter the enzyme structure, potentially leading to the formation of unwanted side products with possible harmful biological effects.^129^ Therefore, optical tools, particularly those operational in the biological transparency window that can offer real-time product fingerprinting of enzyme activity are of paramount importance in the field of disease diagnostics. While this approach offers the advantage of directly screening the product of enzyme activity, challenges may arise from possible limited stability of the product in a biological medium and its preferential reactivity with other chemical species present in complex biological environments. These factors have the potential to compromise the interaction of the product with the SWCNTs, which forms the basis of monitoring enzyme activity.

### Monitoring the Chemical Energy Produced in Enzymatic Reactions

Energy production in enzymatic reactions is a fundamental aspect of cellular metabolism. Enzymes are crucial in converting substrates into products, releasing energy the cell can use for various cellular processes.^130^ This involvement extends to various biochemical processes, including substrate oxidation–reduction reactions, glycolysis, the Krebs cycle, photosynthesis, and chemiosmosis.^131^ The energy generated or consumed in an enzymatic reaction can serve as an indicator of the enzyme activity. For example, an enzymatic activity that produces light emission within the wavelength range corresponding to the absorption of SWCNTs can excite their fluorescence emission. This can provide a versatile platform for monitoring enzymatic reactions without the need for the chemical design of dispersants of SWCNTs, specifically tailored with substrates or designed for interaction with the products. However, a prerequisite is that the energy generated during the enzymatic reaction aligns with the excitation wavelength range of the SWCNTs.

**Figure 7:**
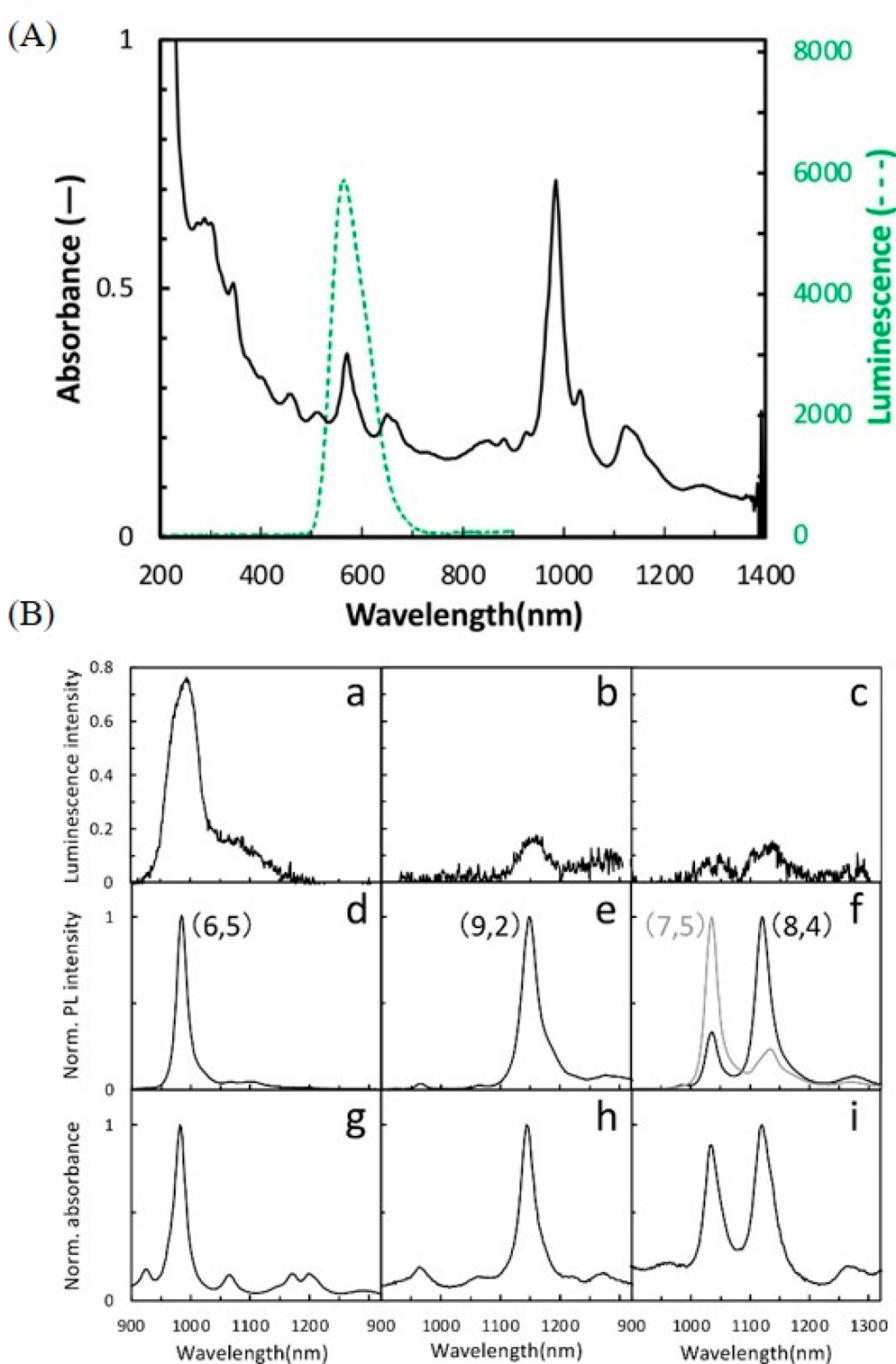
(A) Optical spectra depicting the overlap of the luminescence peak from oxyluciferin (the luciferase reaction product) with the absorption spectrum of DOC-SWCNTs. (B) Luminescence spectra of SWCNTs illuminated by (a–c) the chemical energy produced following luciferase-mediated formation of oxyluciferin and (d, e) an exogenous light source for various chiralities of SWCNTs. (g–i) Corresponding absorption spectra of (6,5), (9,2), and a mixture of (7,5) and (8,4) SWCNTs. Reproduced with permission from ref (105). Copyright 2023 American Chemical Society.

To this end, the Kataura research group observed the fluorescence of deoxycholate (DOC)-stabilized (6,5)-enriched SWCNTs due to luciferase activity, resulting in the conversion of luciferin to oxyluciferin.^105^ Luciferase catalyzes the transformation of luciferin into oxyluciferin, producing excited oxyluciferin (oxyluciferin*) as an intermediate. Upon returning to the ground state, oxyluciferin* emits light with a wavelength of 562 nm, which overlaps with the excitation of the (6,5)-enriched SWCNTs (Figure 7a), leading to observable emission from the DOC-SWCNTs when in proximity to the enzymatic setup (Figure 7b). In addition to the (6,5) chirality, the concept could be extended to other chiralities, including (9,2), and a mixture of (7,5) and (8,4).

The primary advantage of this strategy, particularly relevant for tracking enzyme activity, is that no exogenous light excitation is needed, allowing for real-time screening of enzyme activity along with spatiotemporal information, thereby significantly advancing diagnostics and imaging in deep tissues. Nevertheless, for broad applicability of this strategy, it is important to carefully ensure sufficient spectral overlap between the energy associated with the emission of the product of enzyme activity and the excitation of the SWCNT sample.

### Functionalization of SWCNTs with Target Enzymes

The suspension of SWCNTs with target enzymes, facilitating interaction with externally introduced substrates and resulting in a modulation of the optical signal of the SWCNTs, provides a practical approach for facile monitoring of enzyme activity. In this context, the key requirement for effective enzyme activity monitoring involves exposing the appropriate enzyme groups to the SWCNTs to ensure their proper dispersion. Simultaneously, it is critical to prevent enzyme denaturation during the dispersion process, ensuring that the enzyme remains active for successful monitoring upon subsequent reaction with the substrate. Further, it is crucial that, after suspending the SWCNTs with the target enzyme, the enzyme’s active site remains accessible to the substrate, allowing the enzyme to effectively interact with it.

Previously, SWCNTs suspended with enzymes have been employed to detect the substrates of the dispersant enzyme, rather than explicitly the activity of enzymes constituting the dispersant of SWCNTs.^85,87^ Nonetheless, we envision that the approach of enzyme-assisted SWCNT suspension, designed to monitor enzyme activity upon subsequent interaction with relevant substrates, can be further developed based on the existing studies that have utilized a similar strategy for biomarker substrate monitoring.

**Figure 8:**
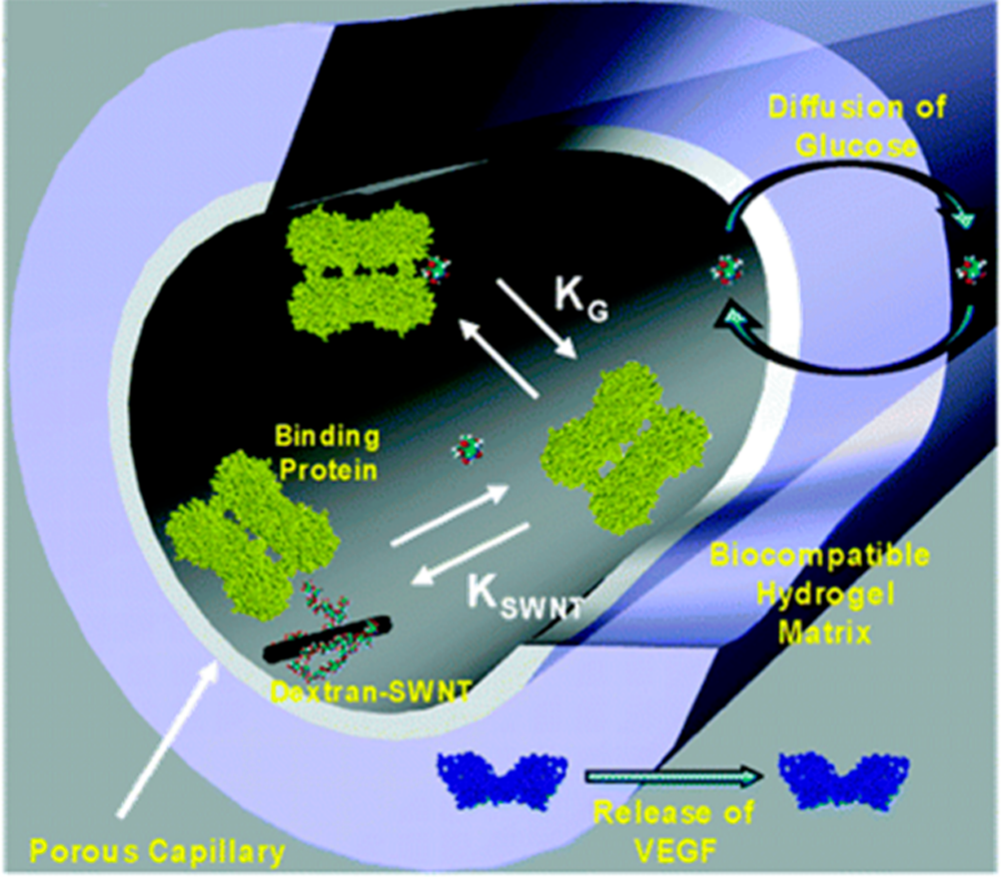
Schematic illustrating the glucose detection mechanism employing dextran suspended SWCNTs. Initially, the fluorescence of dextran-SWCNTs was reduced upon binding of apo-GOx. However, exposure to glucose resulted in the restoration of fluorescence. Reproduced with permission from ref (132). Copyright 2005 American Chemical Society.

Considering this perspective, the Strano research group has demonstrated the dispersion of SWCNTs with glucose-specific proteins like concanavalin A (ConA) and apo-glucose oxidase (apo-GOx), which is the inactive form of GOx, for effective monitoring of glucose.^132^ The SWCNTs were initially suspended by glucose analogues like dextran, which were then bound to apo-GOx. Apo-GOx is the apoenzyme state of GOx, wherein the enzyme lacks the flavin adenine dinucleotide (FAD) cofactor responsible for the catalytic activity. Interestingly, upon binding to apo-GOx, the fluorescence of dextran-suspended SWCNTs significantly decreased. However, following sequential exposure to glucose, the fluorescence of the SWCNTs was restored, providing a means to monitor the presence of glucose (Figure 8). In this particular investigation, as well as in related studies by the Strano research group,^133^ the primary objective was to detect glucose. However, adopting a method wherein GOx-suspended SWCNTs interact with glucose, leading to SWCNT fluorescence modulation, is a promising approach for the direct monitoring of GOx activity.

For example, the Boghossian research group successfully engineered a reversible nanosensor for monitoring glucose using GOx-suspended SWCNTs, circumventing the need for exogenous mediators.^85^ Exposing GOx-SWCNTs to glucose resulted in a significant increase in the fluorescence intensity of the enzyme-stabilized SWCNTs, which was significantly more pronounced than with other saccharides like mannose, galactose, maltose, fructose, and xylose. To unequivocally attribute this fluorescence enhancement to the interaction with glucose, control experiments were conducted by exposing glucose to thermally denatured GOx-dispersed SWCNTs, resulting in no discernible increase in fluorescence. Interestingly, the strategically designed sensors were demonstrated to be stable over a wide range of pH values, from 3 to 7, exhibiting negligible variation in fluorescence intensity with changes in pH. This achievement marks a significant step toward the practical application of these sensors in real-world samples for sensing purposes. The key for the reversibility and the elimination of a mediator in fluorescence-enhancement-based glucose detection was suggested to result from the passivation of electrons transferred from glucose to defect sites of GOx-SWCNTs. The advent of such techniques for effectively monitoring important biomarkers is envisioned to contribute significantly to the further advancement of diagnostics.

Additionally, the Landry research group have successfully developed a biosensor for the reversible monitoring and imaging of glucose in biological fluids and mouse brain slices, utilizing GOx- and apo-GOx-SWCNTs.^87^ GOx-SWCNTs, prepared by directly sonicating SWCNTs with GOx, exhibited immediate enhancement of the fluorescence intensity upon incubation with glucose in a buffer, forming the basis for monitoring glucose levels. Notably, other saccharides such as fructose, galactose, fucose, mannose, xylose, maltose, and sucrose did not significantly increase the fluorescence of GOx-SWCNTs under identical conditions, thereby highlighting the selectivity of the sensors. Also, the apo-GOx-SWCNTs sensors for glucose were found to be stable in human whole serum for up to 3 days, thereby emphasizing the potential of these sensors for long-term in vivo sensing applications. Mechanistic investigations revealed that the underlying principle for monitoring glucose relied on substrate-enzyme binding rather than GOx-mediated oxidation of glucose. Additionally, apo-GOx-SWCNTs were designed to effectively detect and quantify endogenous glucose in plasma and enabled real-time imaging of glucose in tissue samples. The reversible operability of the GOx-based SWCNTs further underscored their potential as tissue-translatable glucose sensors with broad applicability. This study lays the foundation for developing innovative and robust biosensors capable of sensing, imaging, and quantifying essential biomarkers, even in complex biological mediums.

Furthermore, a recent study by the Landry research group showcased the covalent conjugation of horseradish peroxidase (HRP) to the surface of SWCNTs suspended by (GT)_15_ single-stranded DNA (ssDNA), through azide-based triazine chemistry, for real-time sensing and imaging of hydrogen peroxide.^134^ Upon exposure of the HRP-conjugated SWCNTs to hydrogen peroxide, a substantial increase in their fluorescence was observed. Additionally, the HRP-conjugated SWCNTs demonstrated the ability to detect the presence of hydrogen peroxide when immobilized on solid surfaces. This study not only contributes to the understanding of covalent functionalization of SWCNTs but also opens new avenues for the functionalization of SWCNTs with various enzymes using a wide repertoire of chemistry.

While these methods hold enormous potential for monitoring the activity of enzymes that disperse SWCNTs, one must verify that the enzyme retains its natural conformation on the surface of the SWCNTs, as partial denaturation may ultimately compromise the activity of enzymes on exogenously added substrates.

## SWCNTs-Based NIR Probes for Improved Monitoring of Enzymatic Reactions Compared to Conventional Gold Standards

As detailed in preceding sections, NIR emitting SWCNTs exhibit notable advantages as sensors for enzymatic activity compared to traditional spectroscopic probes functioning in UV–visible regions. This is attributed to several unique features of SWCNTs, including a fluorescence emission within the biological transparency spectral region, remarkable photostability, ease of chemical functionalization of their surface, and physicochemical stability in biological mediums, among others. Nonetheless, a comprehensive investigation providing tangible evidence for the enhanced monitoring of enzymatic activity using SWCNTs, surpassing traditional techniques, is crucial to substantiate significant advancements in this field. In this context, it was unequivocally demonstrated that the sensitivity for monitoring CHE enzyme activity, achieved through appropriately functionalized SWCNTs, matched that of the conventional Ellman assay.^99,107^ Beyond this, SWCNTs have additional benefits like spatiotemporal information, real-time monitoring of biological processes, and background-free screening of enzyme activity in complex biological fluids, underscoring their potential over traditional gold standard analogues.

To this end, a recent article by the Kruss research group provides additional support for using structurally intricate and NIR-emitting SWCNTs as a superior tool for monitoring enzyme activity compared to UV–visible-based spectroscopic probes.^103^ The study demonstrated conclusively that enzymatic reactions, typically monitored through optical signals in the UV–vis region, can be tracked with significantly lower limits of detection using a strategy that enables signal amplification with SWCNTs. The study hypothesized that the substrates and products of enzymatic reactions containing aromatic rings can bind to the hydrophobic surface of the SWCNTs through potential π–π interactions, modulating the SWCNT fluorescence based on surface interactions. This binding potentially increases the effective concentration of these analytes around the SWCNTs, enhancing optical signals and thus lowering detection limits for enzymatic transformations. In this study, SWCNTs were suspended by phospholipid-polyethylene glycol (PL-PEG) or two ssDNA sequences, (AT)_15_ and (GT)_15_. The resulting ssDNA-SWCNTs displayed high sensitivity to both the substrate (p-phenylenediamine) and the corresponding product (Bandrowski’s base) of HRP. Specifically, p-phenylenediamine increased SWCNT fluorescence, peaking at 2.5 μM, while Bandrowski’s base completely quenched it at 5 μM. This sensitivity is notably higher than that of traditional UV–vis detection methods like enzyme-linked immunosorbent assay (ELISA), which only detects the product of HRP activity at a 50-fold lower sensitivity. Additionally, to broaden the versatility of the approach, a more common substrate of HRP was also employed to validate the proof-of-concept.

This approach sets a new lower limit for the detection of enzymatic activity, overcoming the limits of conventional techniques like ELISA, which is crucial for real-world enzymatic activity monitoring, by enhancing the local concentration of enzyme substrates and products on the surface of the SWCNTs. This also illustrates a general strategy for real-time monitoring of enzymatic activity in the advantageous NIR region by observing the distinct effects of substrates and products of enzymatic reactions on the optical properties of SWCNTs.

## Monitoring Enzyme Inhibition Using NIR Fluorescent SWCNTs

Screening for enzyme inhibition is a crucial aspect of drug discovery, toxicological studies, understanding disease mechanisms, and gaining insight into cellular metabolism.^135,136^ Many therapeutic drugs operate by inhibiting specific enzymes involved in disease processes.^21^ Monitoring enzyme inhibition is thus essential for identifying potential drug candidates and evaluating their effectiveness. Additionally, assessing whether a specific chemical species inhibits an enzyme provides insights into its potential adverse effects on biological systems. Furthermore, inhibition of seminal enzymes often indicates incipient diseases, thus making it a significant aspect of diagnostic and prognostic research. Thus, developing biosensors that provide real-time information about enzyme inhibition is therefore of paramount importance.

Various techniques employing SWCNT-based probes have been developed to assess enzyme inhibition, leveraging the principles established for enzyme activity monitoring. As detailed previously, the modulation of SWCNT fluorescence, triggered by enzyme activity, serves as a key indicator of active, uninhibited enzymes. Conversely, the presence of inhibitors hampers enzyme activity, resulting in the absence of fluorescence modulation. This lack of change in fluorescence effectively indicates the presence of inhibition, providing a direct measure of the inhibitor’s impact on enzyme functionality.

**Figure 9:**
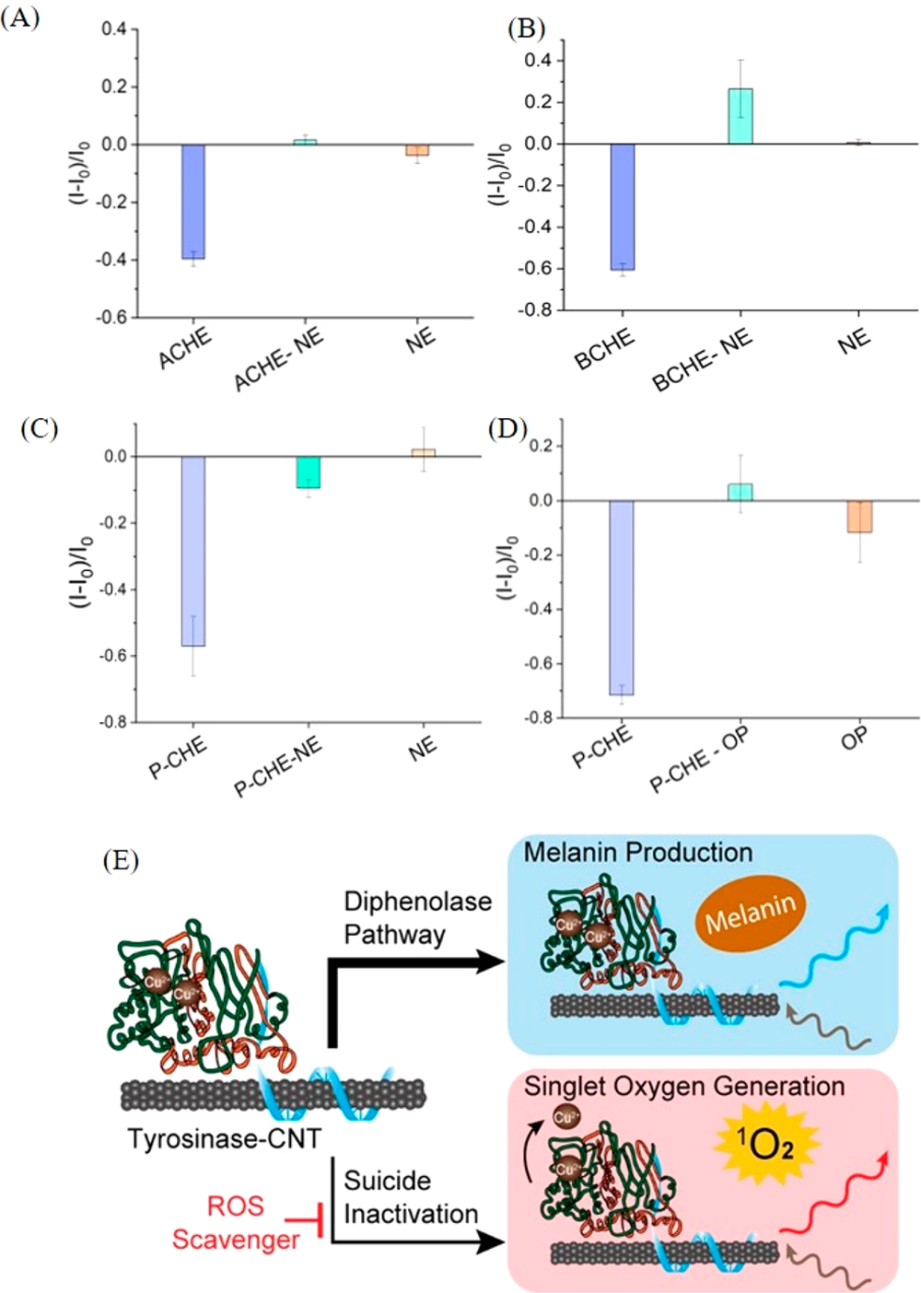
Normalized fluorescence of MC-SWCNTs in response to uninhibited and NE-inhibited (A) ACHE and (B) BCHE. Normalized fluorescence of MC-SWCNTs in response to uninhibited P-CHE and P-CHE inhibited by (C) NE and (D) OP.^107^ Reproduced with permission from ref (107). (E) Schematic highlighting the principle for monitoring the suicide inactivation of tyrosinase enzymes present on the surface of SWCNTs through fluorescence modulation of the latter. Reproduced with permission from ref (102). Copyright 2023 American Chemical Society.

In this regard, it has been reported that MC-SWCNTs could be used to detect the inhibition of CHE enzymes in addition to monitoring their activity^107^ (as already discussed above). MC-SWCNTs were instrumental in monitoring CHE inhibition in buffer and plasma fluids, representing a vital strategy to be used in clinically relevant samples. When incubated with CHE enzymes previously exposed to synthetic inhibitors such as neostigmine bromide (NE), MC-SWCNTs did not show a significant decrease in fluorescence. In contrast, uninhibited CHE under similar conditions exhibited a substantial decrease in MC-SWCNT fluorescence. Additionally, when P-CHE was incubated with inhibitors like organophosphates (OP) commonly found in pesticides,^137^ the enzyme was inhibited, leading to a lack of decrease in the fluorescence of MC-SWCNTs, thereby allowing for the monitoring of enzyme inhibition. This clearly highlighted the application of substrate-suspended SWCNTs for monitoring enzyme inhibition in real-world scenarios (Figure 9A–D). Additionally, the inhibition of ACHE and BCHE using NE could be detected with DNA-SWCNTs.^99^

Adopting a distinct approach, the Heller research group has introduced a SWCNT-based optical probe specifically tailored for monitoring enzymatic suicide inactivation (ESA), an irreversible form of enzyme inhibition.^102^ The fundamental principle for monitoring ESA involved the interaction of products (singlet oxygen) formed during ESA with the enzyme-bound SWCNTs, leading to a bathochromic shift in the fluorescence of the SWCNTs. This technique was designed to directly observe the inactivation process, as opposed to using SWCNT sensors initially designed for monitoring enzyme activity. This study not only furnished real-time data on the extent of ESA using NIR-emitting SWCNTs but also unveiled novel possibilities for employing SWCNTs as probes in drug screening and gaining deeper insights into the mechanisms of enzyme inhibition pathways (Figure 9E).

## Challenges and Future Outlook in Monitoring Enzyme Activity Using SWCNTs

**Scheme 2:**
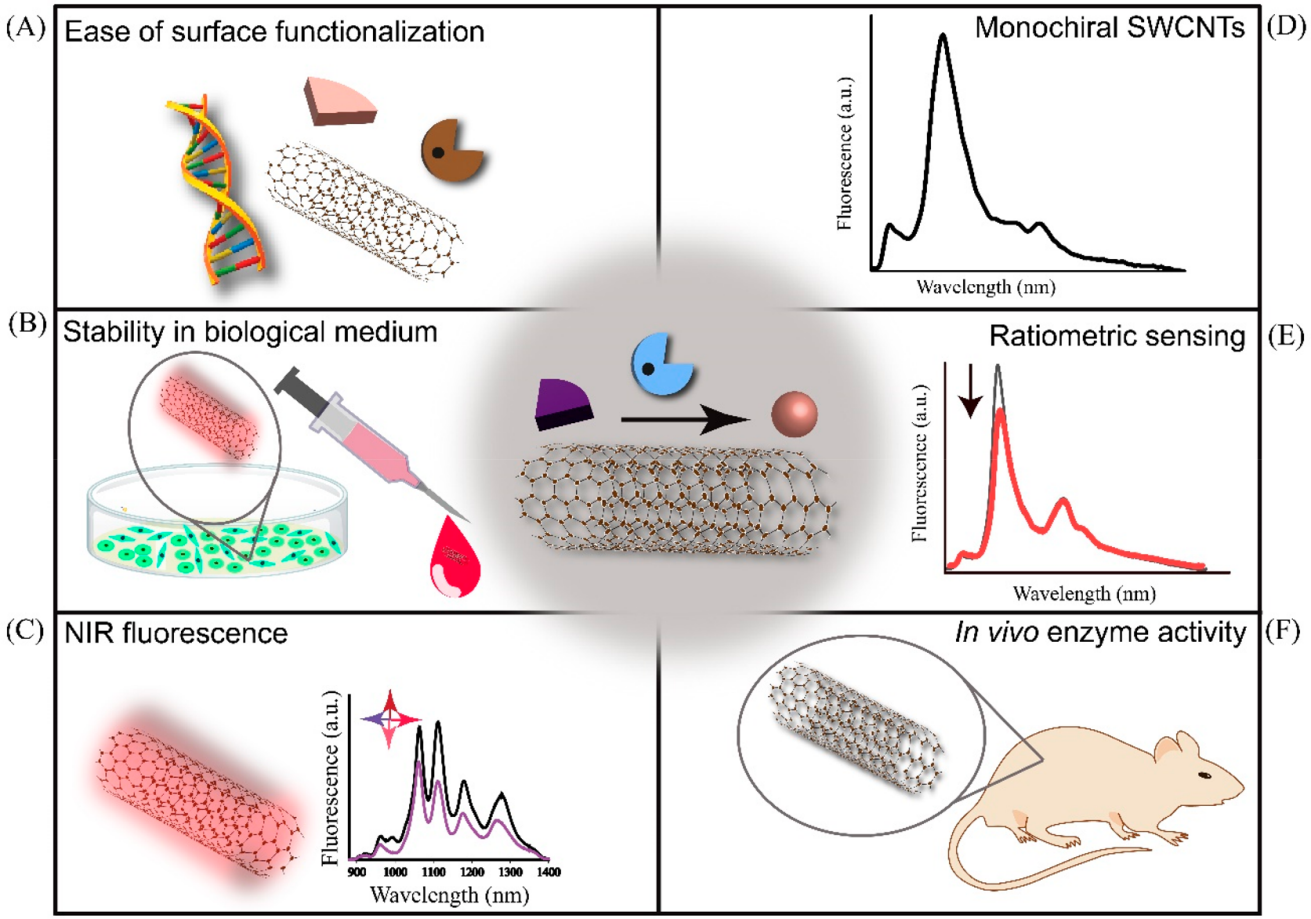
Graphical Overview of the Advantages and Future Opportunities for SWCNT-Based Monitoring of Enzyme Activity SWCNTs benefit from (A) the ease of chemical functionalization of their surface, (B) remarkable physicochemical stability in biological mediums, and (C) fluorescence emission and response within the biological transparent NIR spectral region. Future avenues for monitoring enzymatic activity include (D) utilizing chirality-pure SWCNT samples, enabling (E) ratiometric sensing and internal calibration, and (F) deep-tissue sensing *in vivo*.

While substantial advancements have been made in using SWCNTs for monitoring enzyme activity (Scheme 2A–C), there remain areas ripe for further development, considering the benefits of SWCNTs. A key challenge in employing nanomaterials like SWCNTs lies in their inherent polydispersity, where each nanoparticle varies in structure, impacting its identity and function. This variability complicates quantitative assessments, as analyses typically rely on averaging properties. A promising solution is the use of chirality-pure SWCNTs, which have a rather uniform diameter and, thus, nearly identical optical and physical properties (Scheme 2d). Recent advances in separation techniques, such as aqueous two-phase extraction (ATPE) and chromatography-based approaches, have facilitated the isolation of chirality-pure SWCNTs.^30,138−147^ In parallel, synthesizing SWCNTs with a specific chirality using tailor catalyst particles has shown promising results.^148−152^ These separations or selective growth not only result in SWCNTs with distinct optical properties, characterized by defined fluorescence emission, but also yield samples of SWCNTs with a rather uniform distribution of lengths, enhancing the reliability of their quantification as enzymatic sensors.^30^

The ability to separate SWCNTs with specific chirality provides further opportunity for ratiometric screening of enzymatic activity using two or more SWCNTs of isolated chirality, either mixed in dispersion or chemically conjugated, benefiting from internal sensor calibration (Scheme 2e). Additionally, this approach mitigates the impact of environmental fluctuations, variations in detector response, optical path length, and local concentration of probes. Therefore, screening enzymatic activity using a ratiometric approach, involving chirality-pure SWCNTs assembled through physical forces or chemical conjugation, holds the potential to provide a more quantitative and reliable method compared to current techniques. It is crucial that not all emission peaks resulting from different chiralities display similar fluorescence modulations resulting from the enzymatic reactions. Ideally, at least one peak remains stable, serving as a reference for internal calibration standards.

Further exploration into defect-induced SWCNTs presents additional possibilities in parallel to ratiometric sensing. Defects in SWCNTs can introduce a new peak corresponding to E_11_* transitions alongside the standard E_11_ transition peak.^153−160^ Consequently, the presence of two distinct peaks in a single SWCNT species opens the possibility for ratiometric probing of enzymatic reactions. Also, in this case, it is imperative that the progression of the enzymatic reaction affects differently the emission characteristics of the E_11_ or E_11_* peaks, allowing for internal standard.

The current state-of-the-art reports the successful monitoring of enzyme activity in both buffers and complex biological media, such as fetal bovine serum and human blood plasma. Looking ahead, the goal is to achieve real-time tracking of enzyme activity in tissues and tumors *in vivo* with enhanced spatiotemporal resolution, fully harnessing the potential of SWCNTs as label-free and background-free sensors (Scheme 2f). Achieving this would necessitate SWCNTs-based sensors to be highly specific to enzyme activity, exempted from local background interference, physiochemically stable in biological media to retain their optical activity, extremely sensitive to minimal enzymatic activity, and capable of providing high throughput readout of optical signals.

In the realm of healthcare, SWCNTs have gained attention for their potential in detecting enzyme activity. However, it is beneficial to extend this focus to monitoring enzymes crucial for industrial biocatalysis. Recent studies have utilized techniques like deep UV resonance Raman spectroscopy to track the activity of industrially significant enzymes such as nitrile hydratase and xanthine oxidase.^161^ Additionally, the successful monitoring of biofilm extracellular polymeric substances degradation by hydrolases further highlights the versatility of monitoring enzyme activity in diverse industrial applications.^125^ Thus, advancing the use of SWCNTs for tracking the activity of industrially relevant enzymes not only holds the potential to optimize biocatalytic processes but also contributes to enhancing productivity and promoting sustainable industrial practices.

## Conclusion

In this perspective, we have provided a detailed exploration of recent advancements in monitoring the activity of a diverse array of enzymes through the utilization of probes based on NIR fluorescent SWCNTs, where the enzyme activity translates to a modulation of the SWCNT fluorescence. We thoroughly explored the distinctive properties of SWCNTs that make them ideal candidates for screening enzymatic activity. Our discussion encompassed a comprehensive overview of various strategies developed for tracking enzyme activity, emphasizing the underlying principles and the necessary conditions for successful implementation. Furthermore, we detailed the diverse approaches devised for screening enzyme inhibition, a pivotal aspect in health and disease monitoring.

The remarkable potential of the application of SWCNT-based assays has been demonstrated in real-world, clinically relevant samples. Moreover, recent studies have underscored the enhanced capabilities of SWCNT-based optical probes in the NIR range over conventional spectroscopic probes operating in the UV–visible region. Nevertheless, we have identified several challenges faced in the development of SWCNT-based probes for monitoring enzyme activity and proposed plausible solutions, setting a course for future innovation in this field and further exploration and refinement of enzymatic assays using SWCNT technology. The potential of SWCNTs in transforming biosensing and diagnostics is vast, and continued exploration in this domain holds promise for groundbreaking discoveries and applications.
